# Resektion einer submakulären Traktionsmembran bei ausgeprägter Visusminderung: Fallbericht

**DOI:** 10.1007/s00347-020-01135-0

**Published:** 2020-06-05

**Authors:** A. Rüger, A. Eichhorst, R. Wienrich, U. Siebolts, M. Fiorentzis

**Affiliations:** 1 Universitätsklinikum Halle (Saale), Klinik und Poliklinik für Augenheilkunde, Martin-Luther-Universität Halle-Wittenberg, Ernst-Grube-Str. 40, 06120 Halle (Saale), Deutschland; 2 Universitätsklinikum Halle (Saale), Institut für Pathologie, Martin-Luther-Universität Halle-Wittenberg, Halle (Saale), Deutschland

**Keywords:** Vitreoretinale Chirurgie, Subretinale Resektion, Subretinale Membran, Durchgebrochene CNV, PEHCR, Vitreoretinal surgery, Subretinal resection, Subretinal membrane, Choroidal neovascular membrane, PEHCR

## Abstract

Eine 89-jährige Patientin stellte sich mit akuter Visusminderung am linken Auge vor. Bei der Fundoskopie zeigten sich große Zellen im Glaskörperraum. Es wurde eine Therapie bei Verdacht auf ein intraokuläres Lymphom begonnen. Bei der Pars-plana-Vitrektomie bei einer Ablatio retinae fand sich kein Anhalt für einen lymphoproliferativen Prozess im histologischen Präparat der Retinektomie nach Resektion einer subretinalen Traktionsmembran. Die Diagnose einer durchgebrochenen chorioidalen Neovaskularisation (CNV) mit subretinaler Membranbildung („peripheral exsudative hemorrhagic chorioretinopathy“ [PEHCR]) wurde gestellt. Es zeigte sich kein Anhalt für Malignität. Die Resektion einer subretinalen Traktionsmembran kann eine Herausforderung für den vitreoretinalen Chirurgen darstellen, bietet aber die Möglichkeit einer Visusbesserung.

## Abstract (en)

An 89-year-old female presented with acute visual impairment and large cells in the vitreous cavity of the left eye. The patient was treated for a suspected intraocular lymphoma. In the follow-up a retinal detachment was diagnosed and treated via pars plana vitrectomy. A resection of the subretinal traction membrane following retinectomy was performed. The histological findings did not reveal any indications of a lymphoproliferative process. A ruptured choroidal neovascularization (CNV) with peripheral exudative hemorrhagic chorioretinopathy (PEHCR) and subretinal membrane formation was diagnosed. There were no signs of malignancy. The resection of a subretinal traction membrane poses a challenge for the vitreoretinal surgeon but can provide the possibility of visual improvement.

## Falldarstellung

### Anamnese

Eine 89-jährige pseudophake Patientin stellte sich mit akuter Visusminderung am linken Auge in unserer Klinik vor. Der Visus war bis auf Handbewegung reduziert. Am Partnerauge lag der Visus bei 0,6.

### Klinischer Befund

Im Glaskörper des linken Auges fanden sich Zellen und eine periphere weißliche chorioretinale Raumforderung. Das rechte Auge zeigte vereinzelte Drusen und einen altersentsprechenden Fundus. Der vordere Augenabschnitt war beidseits regelrecht und reizfrei.

### Diagnostik

In der optischen Kohärenztomographie (OCT) zeigten sich beidseits weiche Drusen bei sonst unauffälligem Fundusbefund. Die Fluoreszenzangiographie war aufgrund des reduzierten Einblicks am betroffenen Auge nicht verwertbar.

#### Serologie/Zytologie.

Die Serologie ergab keinen Anhalt für eine akute virale Infektion, eine Borreliose oder Syphilis. Die Liquorzytologie zeigte eine lymphozytäre Pleozytose mit suspektem Befund hinsichtlich eines lymphoproliferativen Prozesses. Diese war in der Immunzytochemie eher lymphoiden Reizformen zuzuordnen.

#### Glaskörperpunktat.

Eine Pars-plana-Vitrektomie mit vitrealer Probengewinnung wurde durchgeführt. Die virologischen Proben des Glaskörperpunktats zeigten keinen HSV-/VZV- oder CMV-Nachweis. Die zytologische Probe war nicht aussagekräftig.

### Therapie und Verlauf

Bei Verdacht auf ein intraokuläres Lymphom wurde die Patientin stationär zur Abklärung aufgenommen. Es wurde eine Pars-plana-Vitrektomie mit vitrealer Probengewinnung, Silikonölendotamponade sowie Endolaserkoagulation bei intraoperativ festgestellten peripheren Ischämien durchgeführt.

In Zusammenschau der Befunde wurde von einem intraokulären Lymphom ausgegangen und als therapeutischer Versuch 0,1 mg Methotrexat (MTX) und 0,1 mg Dexamethason intravitreal appliziert. Dies entspricht jeweils der Reduktion auf ein Viertel der Standarddosis der Medikamente unter Silikonölendotamponade. Hierunter stieg der Visus an, weshalb weitere intravitreale Injektionen ambulant geplant wurden.

**Figure 1:**
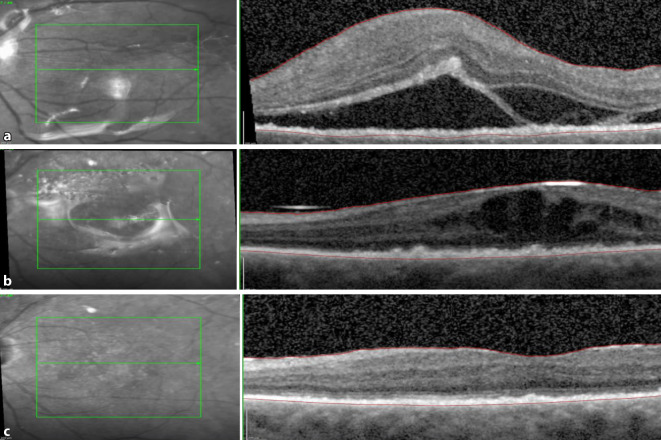

**Figure 2:**
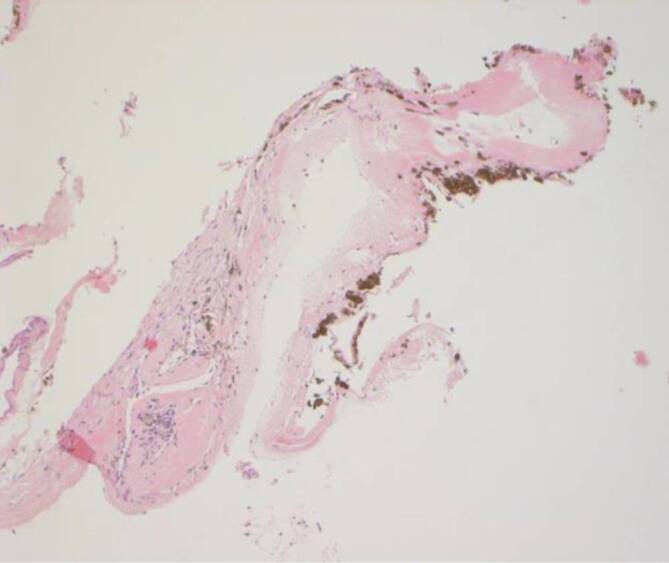

**Figure 3:**
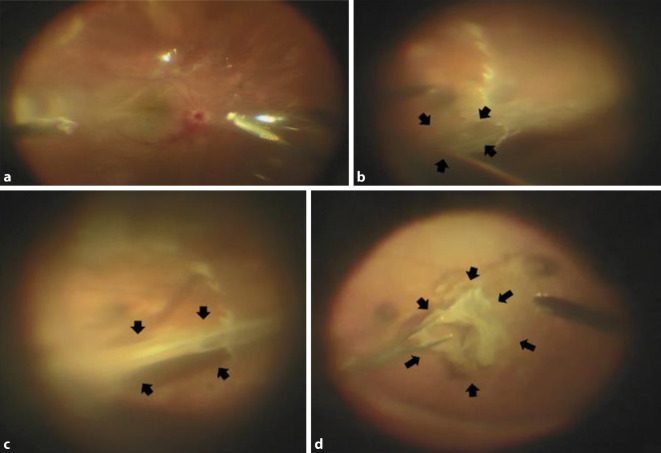

Nach 4‑maliger intravitrealer MTX- und Dexamethason-Injektion zeigte sich zur Wiedervorstellung eine traktive Ablatio retinae unter Silikonölendotamponade (Abb. 1a). In der präoperativen optischen Kohärenztomographie (OCT) stellte sich eine subretinale Membran dar (Abb. 2). Intraoperativ stellte sich eine ausgeprägte subretinale Traktionsmembran (Abb. 2) mit einer Gesamtgröße von ca. 16 Papillenflächen dar. Sie erstreckte sich von der Papille über die Makula bis zu den großen Gefäßbögen (Abb. 3a–d). Eine Retinektomie wurde peripher zur Aderhaut/Netzhaut-Probengewinnung durchgeführt. Dadurch wurde die Membran vorsichtig mobilisiert und mit zarten wellenartigen Bewegungen von ihren peripheren Ansätzen abgelöst. Nachdem sich die Membran zusammengerollt hatte, blieb ein Anhang an der Papille fest, welcher mittels Traktion befreit wurde.

Nach Entfernung der subretinalen Traktionsmembran entspannte sich die Netzhaut deutlich und legte sich unter Perfluorcarbon (PFCL) wieder an. Anschließend wurde eine weitere Laserkoagulation durchgeführt.

Histopathologisch und durchflusszytometrisch konnten weder bei dem Aderhaut/Netzhaut-Punktat noch bei dem subretinalen Membranpräparat maligne Zellen nachgewiesen werden, weshalb auf weitere intravitreale Injektionen verzichtet wurde. Zur Wiedervorstellung 6 Wochen postoperativ zeigten sich eine deutliche Visusbesserung auf cc 0,2 und zirkuläre Netzhautanlage (Abb. 1b). Bei der ambulanten Kontrolle nach Silikonölentfernung stieg der Visus auf cc 0,5 an (Abb. 1c und 4).

**Figure 4:**
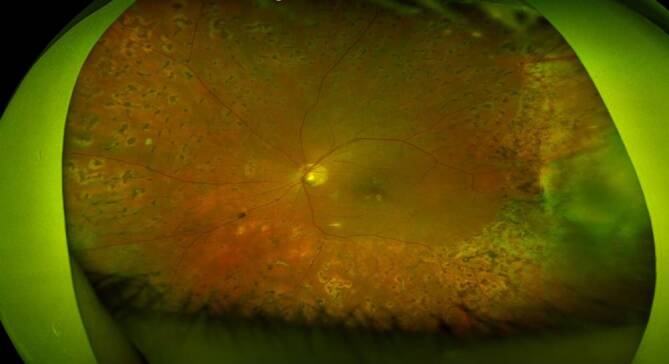

## Diskussion

Bei fehlendem Malignitätsnachweis wurde von einer durchgebrochenen peripheren CNV mit subretinaler Membranbildung ausgegangen. Diese Diagnose stützte sich auf den intraoperativen Befund und den Ausschluss weiterer Differenzialdiagnosen wie einer infektiösen Genese.

Wu et al. berichteten über 22 Patienten im Alter von 50 bis 85 Jahren mit klassischen CNV-Membranen und einer subretinalen Resektion. Die Größe der Membranen lag zwischen 1 und 6,5 Papillenflächen. Bei 10 Augen zeigte sich im Median nach 12,5 Wochen postoperativ eine Visusbesserung (>3 Snellen-Reihen). In keinem Fall kam es zu einer Visusverschlechterung. Im Rahmen des durchschnittlichen Follow-up von 37 Monaten zeigten 59 % ein Rezidiv [1]. Die PEHCR ist eine degenerative Erkrankung der Netzhaut und Aderhaut, die häufig ältere Patienten betrifft [2]. Prognostisch relevant scheinen die postoperative Integrität des retinalen Pigmentepithels nach Entfernung der Membran sowie die Genese der CNV zu sein. In der Studie von Berger et al. zeigte sich dabei lediglich eine leichte Visusbesserung oder Stabilisation des Befundes [3]. In diesem Zusammenhang ist die Besserung des postoperativen Visus bei der vorgestellten Patientin beeindruckend. Der Visusanstieg bei unserer Patientin ist sowohl auf die Resektion der subretinalen Traktionsmembran als auch auf die dadurch ermöglichte Wiederanlage der Netzhaut zurückzuführen. Weiterhin muss betrachtet werden, dass in vielen Fällen im Follow-up eine Rezidivmembran oder Verschlechterung des Visus z. B. bei geografischer Atrophie oder zystoidem Makulaödem einsetzte, was bei unserer Patientin bisher nicht beobachtet wurde [1, 3, 4]. Laut Seibel et al. sind in ca. der Hälfte der Patienten mit PEHCR makuläre Veränderungen in Form von Drusen bis hin zur exsudativen AMD zu erkennen [5]. Die Resektion einer subretinalen Traktionsmembran kann aufgrund ihrer Komplexität und der damit assoziierten Komplikationen eine Herausforderung für den Operateur darstellen. Jedoch offeriert sie eine operative Option bei schweren Fällen und bietet für den Patienten die einzige Möglichkeit einer Visusbesserung.

## Fazit für die Praxis

Bei suspekten zytologischen Befunden sollte neben der Differenzialdiagnose eines lymphoproliferativen Prozesses auch an eine ältere durchgebrochene CNV mit Glaskörperzellen und subretinaler Membranbildung gedacht werden. Die Resektion auch älterer subretinaler Membranen v. a. im Makulabereich kann, auch bei initial schlechter Prognose, eine deutliche Visusbesserung bewirken.

Bei retinalen Infiltraten und Glaskörperzellen sollten auch seltene Differenzialdiagnosen in Betracht gezogen werden, und auch bei scheinbar schlechter Visusprognose sollte eine chirurgische Intervention nicht gescheut werden.
